# High-Grade Round Cell Neoplasm Presenting as an Anterior Mediastinal Mass With Features of Desmoplastic Round Cell Tumour

**DOI:** 10.7759/cureus.98309

**Published:** 2025-12-02

**Authors:** Maridas T Thomas

**Affiliations:** 1 General Internal Medicine, University Hospitals of Leicester, Leicester, GBR

**Keywords:** anterior mediastinal mass, chemotherapy, desmoplastic small round blue cell tumours, rare tumour, surgery, young female

## Abstract

Desmoplastic small round cell tumours (DSRCT) are highly aggressive neoplasms typically affecting adolescent and young adult males. We present a rare case of high-grade round cell tumour with features of DSRCT in a young female who initially presented with anaphylactic reactions to blueberries, which, on further evaluation, revealed an anterior mediastinal mass. Contrast-enhanced CT (CECT) of the thorax demonstrated a large, round cell tumour encasing multiple mediastinal structures, later confirmed by histopathology. The patient was offered combined chemotherapy and radiotherapy; however, she opted to leave against medical advice. This case highlights an unusual presentation of DSRCT and underscores the importance of early recognition and multidisciplinary management.

## Introduction

Round cell tumours are aggressive neoplasms requiring early diagnosis and prompt treatment for improved survival. Round cell tumours encompass a wide variety of tumours which show features of round to medium-sized cells with scanty cytoplasm and hyperchromatic nuclei. The various types of tumours can be distinguished by the variety of clinical presentation, tumour markers and the radiological features. They exhibit a strong male predominance. Desmoplastic small round cell tumour (DSRCT), a subtype of this group, is characterized by nests of small, round tumour cells surrounded by desmoplastic stroma. It most commonly occurs in the abdomen, with thoracic presentations being exceedingly rare. Here we describe a rare case of small round cell tumour which presented as an anterior mediastinal mass and shows clinical and histopathological features of a round cell tumour and is mostly suggestive of a DSRCT, though confirmation without doubt would require an immunohistochemistry showing expression of epithelial, mesenchymal and neural markers.

## Case presentation

A 20-year-old female presented with a two-month history of cough, intermittent facial swelling, hoarseness of voice, and wheezing. Initially, these symptoms were attributed to an allergic reaction to blueberries, which she frequently consumed. During two prior hospital visits, she was diagnosed with anaphylaxis and treated successfully with adrenaline and corticosteroids. Although her symptoms improved temporarily upon discontinuation of blueberries, she noted progressive cough, distension of neck veins, and worsening hoarseness, all of which were suggestive of a superior vena cava syndrome.

**Figure 1:**
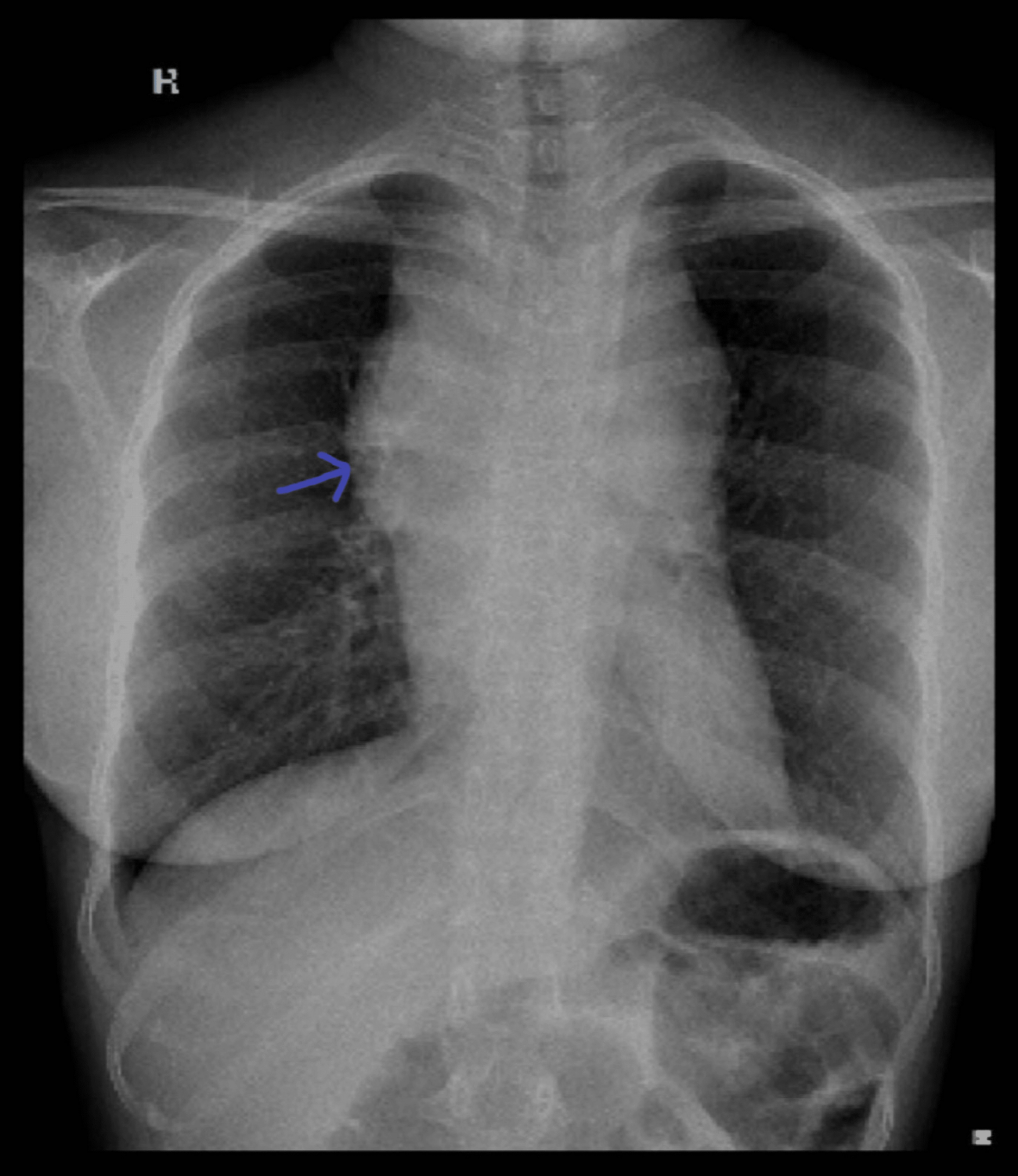
Chest X-ray posteroanterior view of chest X-ray showing features of a mediastinal mass, more prominent over the right side, widening of the superior mediastinum, and obscuration of the right cardiac border.

**Figure 2:**
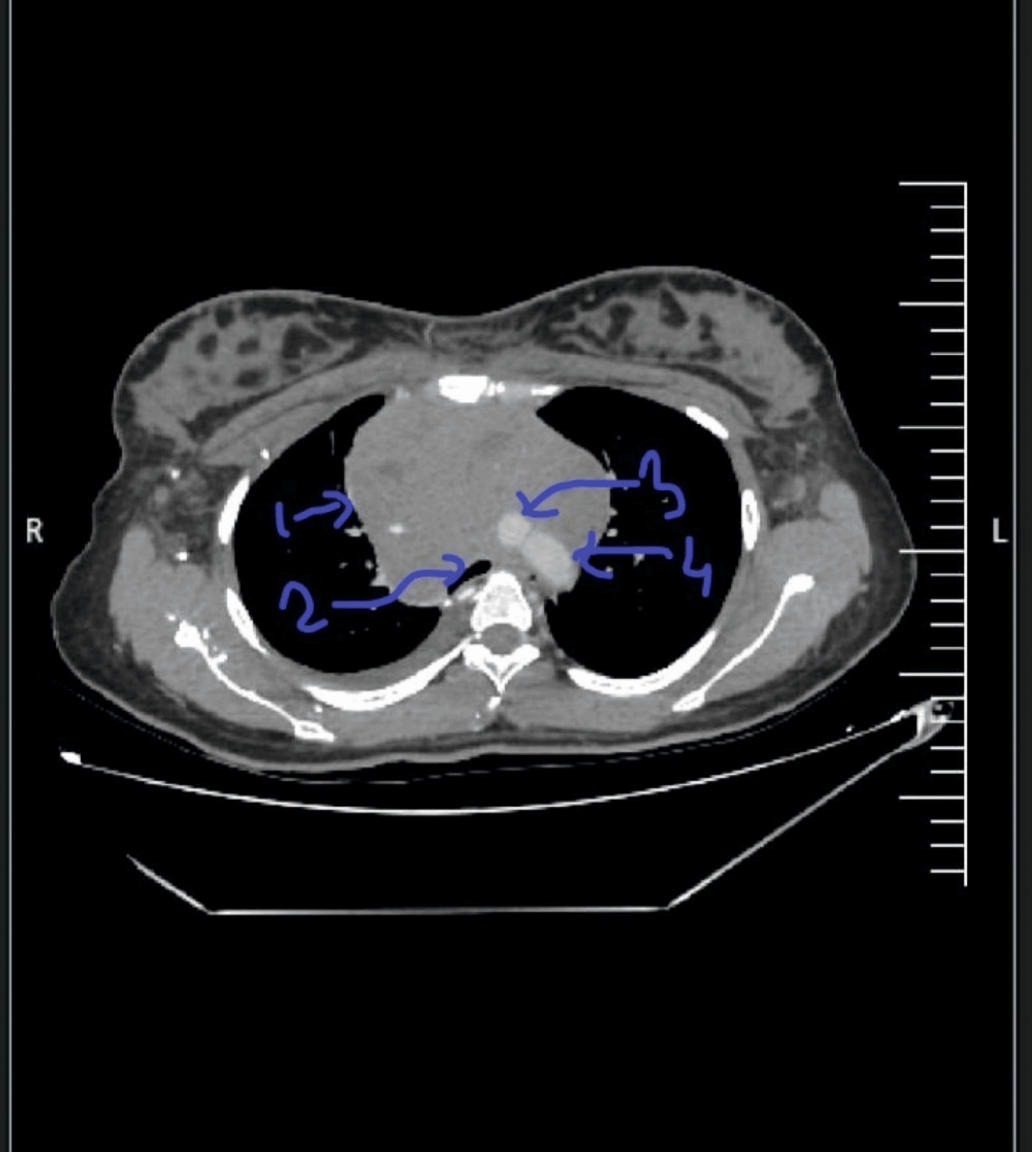
CECT of the thorax showing an anterior mediastinal mass CECT of thorax showing anterior mediastinal mass (1) almost completely encasing aorta (3), Superior vena cava (4), and partially encasing right atrium, oesophagus and trachea (2).

On admission, a chest X-ray revealed an anterior mediastinal mass (Figure 1). Laboratory evaluation showed elevated lactate dehydrogenase (LDH) levels, raising suspicion for lymphoma. Other tumour markers like Alpha Feto Protein and Beta hCG were within normal levels. Contrast-enhanced computed tomography (CECT) of the thorax revealed an ill-defined, heterogeneously enhancing mass measuring 83 × 108 × 103 mm in the prevascular region of the anterior mediastinum, predominantly on the right side (Figure 2). The lesion abutted the right atrium with loss of fat planes posteriorly and contained necrotic and cystic areas. It encased the superior vena cava and branches of the aortic arch, with partial encasement of the trachea and oesophagus, causing mild compression. Multiple enlarged supraclavicular (up to 13 × 11 mm) and mediastinal lymph nodes (up to 15 × 10 mm) were noted, suggesting possible metastasis.

**Figure 3:**
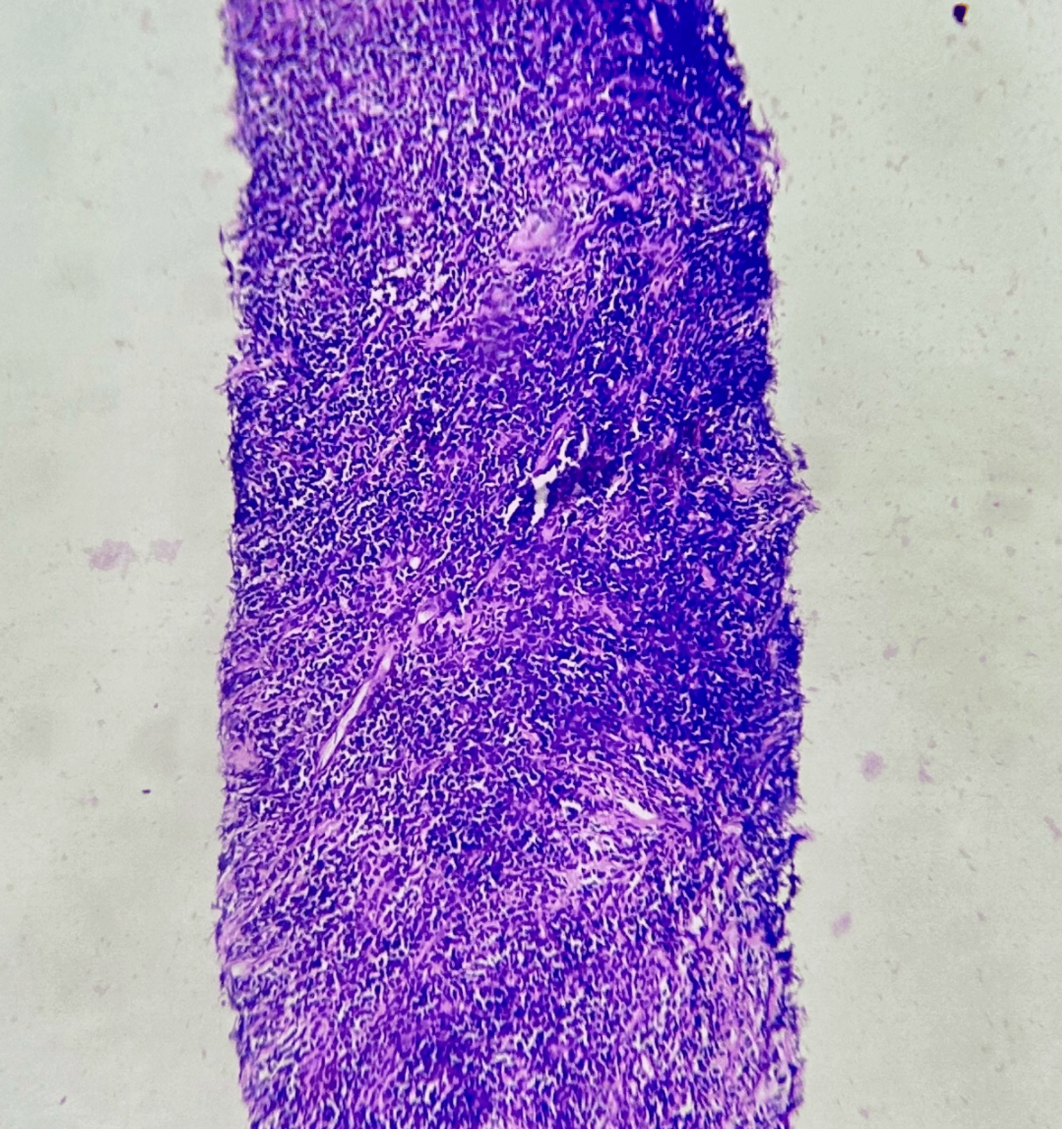
Tru-cut biopsy of the anterior mediastinal mass (Low Magnification) Linear core of cellular neoplasm arranged in diffuse sheets.

**Figure 4:**
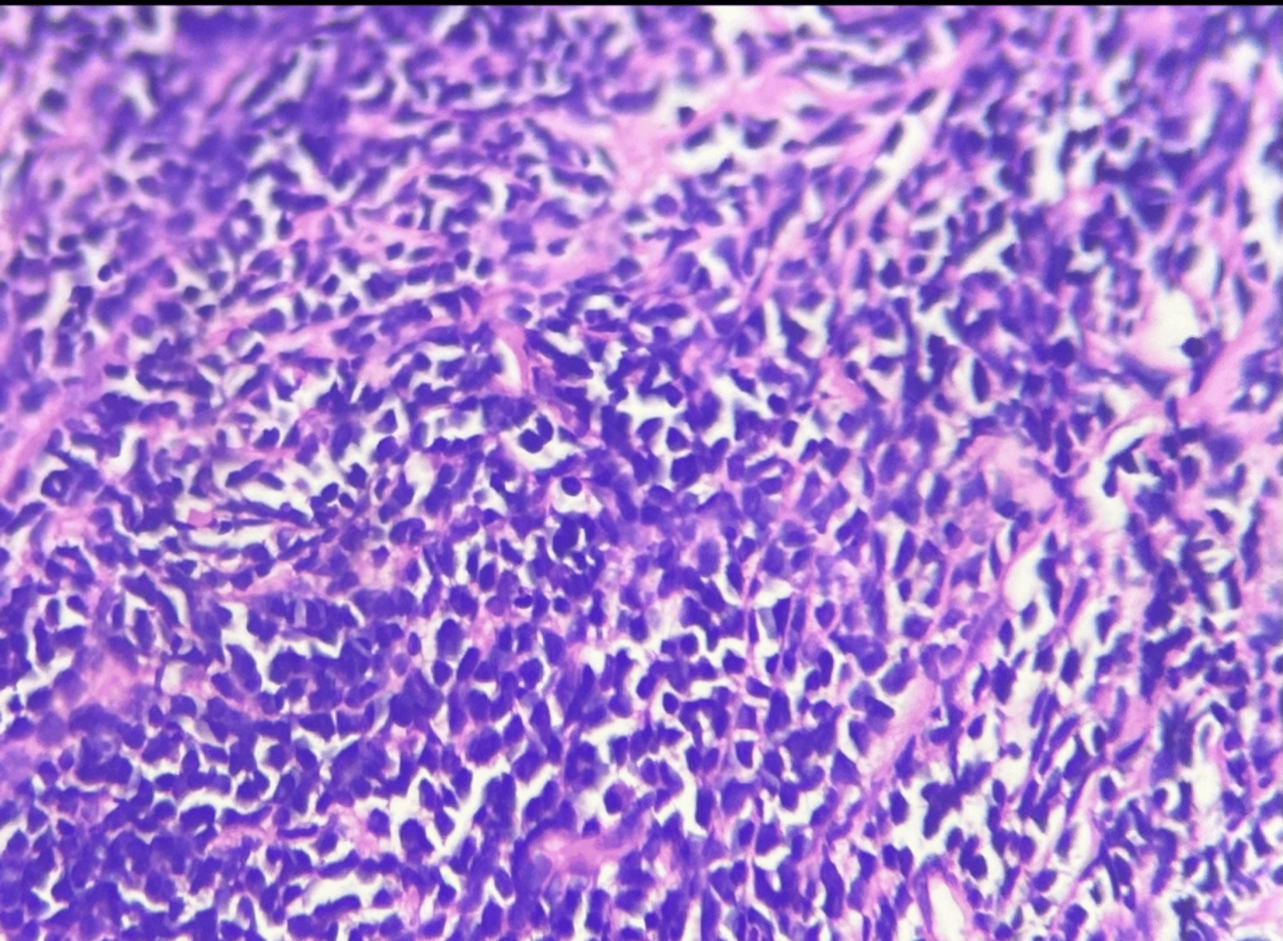
Tru-cut biopsy of the anterior mediastinal mass (High Magnification) Small to medium-sized round and uniform cells with scanty cytoplasm and hyperchromatic nuclei embedded within a fibrous stroma (desmoid), which is suggestive of a small round cell tumour, mostly in favour of a DSRCT DSRCT: desmoplastic small round cell tumours

A tru-cut biopsy demonstrated sheets of medium-to-large uniform round cells with scant cytoplasm, hyperchromatic nuclei, and intervening sclerotic fibrous bands, consistent with a round cell neoplasm (Figures 3-4). Immunohistochemistry (IHC) was advised, but declined by the patient. Due to the tumour’s high-grade nature and involvement of major mediastinal vessels, surgical resection was deemed infeasible. The case was discussed in a multidisciplinary tumour board, and combined chemoradiotherapy was planned. However, the patient chose to leave the hospital against medical advice to seek further opinions: differential diagnosis; non-seminomatous germ cell tumour; lymphoma; thymoma.

## Discussion

Our patient presented with an anterior mediastinal tumour with histology suggestive of a round cell tumour. The detailed histology of the patient did show features of a desmoplastic round cell tumour, such as small, round and uniform cells in a fibrous stroma, though we require an immunohistochemistry to confirm the same. Other neoplasms which can present as anterior mediastinal masses were considered. But normal AFP and beta hCG levels ruled out germ cell tumour, and the histology was not suggestive of lymphoma. The mass was not lobulated as usually seen in Thymomas. Desmoplastic round cell tumours (DSRCT) are rare neoplasms with high-grade malignant potential, most commonly reported among young white males. The condition was first described in 1989 [1]. It is usually found in the abdomen, with common sites including the posterior peritoneum, pelvic cavity, omentum, and mesentery. Less commonly, it has been described in other organs such as the testes, ovaries, and liver. Thoracic involvement is rare, although occasional cases have been reported in the lungs and pleura [2]. Patients typically present with abdominal pain, an abdominal mass, or abdominal distension, and in some cases with features of intestinal obstruction [3-6]. When the lungs are involved, symptoms such as cough and chest tightness are common. The male-to-female ratio of presentation ranges between 4:1 and 10:1, with a median age of diagnosis between 19 and 22 years [7,8]. Hormonal differences are believed to contribute to this gender disparity. Cases of desmoplastic round cell tumours presenting as anterior mediastinal masses are extremely rare. Histologically, round cell tumour cells are small to medium in size, round or oval in shape, and have scant cytoplasm, indistinct borders, deeply basophilic nuclei, and inconspicuous nucleoli [9]. DSRCT belongs to the broader family of small round cell neoplasms of childhood, which includes embryonal rhabdomyosarcoma, poorly differentiated synovial sarcoma, and primitive neuroectodermal tumour. In some intra-abdominal cases, tumour markers such as CA-125 and neuron-specific enolase (NSE) may be elevated, occasionally leading to a misdiagnosis of ovarian carcinoma; however, these markers are not typically elevated in mediastinal tumours [10]. The most common diagnostic modalities include CT and histopathological examination. DSRCT is an aggressive malignancy that can occur in either gender, though literature consistently reports a clear male predominance. Patients may present with variable clinical features and at atypical sites, as seen in the rare mediastinal presentation. Early imaging and tissue diagnosis are essential, as the tumour is highly aggressive and tends to rapidly involve multiple organs and structures. Treatment typically involves a multimodal approach comprising chemotherapy, radiotherapy, and surgical resection. Management should be individualized, taking into account the stage of disease, patient age, comorbidities, and the extent of local invasion.

## Conclusions

This case report emphasizes the possibility of an anterior mediastinal tumour in a young female, which could have been dismissed if not for radiological imaging. The presumptive diagnosis of desmoplastic round cell tumour after discussion with expert pathologists. DSRCT is a rare and aggressive malignancy that can occur at atypical sites and mimic benign conditions. This case underscores the need for a high index of suspicion in patients with unusual or persistent symptoms and mediastinal masses, regardless of gender. Early diagnosis, multidisciplinary management, and patient compliance with therapy are critical for improving outcomes.

## References

[1] Gerald WL, Rosai J (1989). Desmoplastic small cell tumour with divergent differentiation. Pediatr Pathol.

[2] Nayak HK, Vangipuram DR, Sonika U, Kar P, Kumar N, Kapoor N (2011). Mediastinal mass - a rare presentation of desmoplastic small round cell tumour. BMJ Case Rep.

[3] Chang F (2006). Desmoplastic small round cell tumors: Cytologic, histologic, and immunohistochemical features. Arch Pathol Lab Med.

[4] Su MC, Jeng YM, Chu YC (2004). Desmoplastic small round cell tumor of the kidney. Am J Surg Pathol.

[5] Bismar TA, Basturk O, Gerald WL, Karl S, Volkan AN (2004). Desmoplastic small cell tumor in the pancreas. Am J Surg Pathol.

[6] Finke NM, Lae ME, Lloyd RV, Gehani SK, Nascimento AG (2002). Sinonasal desmoplastic small round cell tumor: A case report. Am J Surg Pathol.

[7] Arora VC, Price AP, Fleming S, Sohn MJ, Magnan H, LaQuaglia MP, Abramson S (2013). Characteristic imaging features of desmoplastic small round cell tumour. Pediatr Radiol.

[8] Kis B, O’Regan KN, Agoston A, Javery O, Jagannathan J, Ramaiya NH (2012). Imaging of desmoplastic small round cell tumour in adults. Br J Radiol.

[9] Wang LL, Ji ZH, Gao Y, Chang H, Sun PP, Li Y (2021). Clinicopathological features of desmoplastic small round cell tumors: Clinical series and literature review. World J Surg Oncol.

[10] Fizazi K, Farhat F, Theodore C, Rixe O, Le Cesne A, Comoy E, Le Chevalier T (1997). CA125 and neuron-specific enolase (NSE) as tumour markers for intra-abdominal desmoplastic small round-cell tumours. Br J Cancer.

